# Management strategies for patients with placenta accreta spectrum disorders who underwent pregnancy termination in the second trimester: a retrospective study

**DOI:** 10.1186/s12884-018-1935-6

**Published:** 2018-07-11

**Authors:** Ran Cui, Menghui Li, Junli Lu, Huimin Bai, Zhenyu Zhang

**Affiliations:** Department of Obstetrics and Gynecology, Beijing Chao-Yang Hospital, Capital Medical University, No.8, North Road of Workers Stadium, Chaoyang District, Beijing, 100020 China

**Keywords:** Adjuvant treatments, Conservative management, Leaving the placenta in situ, Placenta accreta spectrum disorders, Pregnancy termination in the second trimester

## Abstract

**Background:**

The unique clinical features of pregnancy termination in the second trimester with concurrent placenta accreta spectrum (PAS) disorders place obstetricians in a complex and delicate situation. However, there are limited data on this rare and dangerous condition. The objective of this research was to investigate and evaluate the clinical management strategies of this patient group.

**Methods:**

The medical records of patients who were diagnosed and treated in our hospital from December 2005 and December 2015 were retrospectively reviewed.

**Results:**

A total of 29 patients were included in this analysis. A prenatal diagnosis was suspected in 8 (27.6%) patients, and the remaining 21 (72.4%) patients were diagnosed after pregnancy termination in the second trimester. In the subgroup with a prenatal diagnosis, a planned hysterotomy was performed in 7 patients who had total placenta previa and previous cesarean delivery. The remaining patient received medical termination. A subtotal hysterectomy was performed in 3 (10.3%) patients for life-threatening bleeding during hysterotomy, and the uterus was preserved with an in situ placenta in the remaining 5 patients. In the subgroup with a postnatal diagnosis, the implanted placenta remained partly or completely in situ in all 21 patients under informed consent. Ultimately, the implanted placenta remained partly or completely in situ in 26 (89.7%) patients in the two subgroups. With the application of adjuvant treatments, including uterine artery embolization and medication followed by curettage under ultrasound guidance, the implanted placenta was passed 76.6 (range: 19 to 192) days after termination. Uterus preservation was achieved in all 26 patients. The complications associated with conservative management included delayed postnatal hemorrhaging (2 cases, 7.7%), fever (6 cases, 23.1%), G1 transaminase disorder (4 cases, 15.4%), and myelosuppression (1 case, 3.8%). Seven women (26.9%) had a spontaneous pregnancy after conservative management, and no patient experienced recurrent PAS disorders.

**Conclusions:**

Leaving the implanted placenta in situ is the preferred choice for patients with PAS disorders who underwent pregnancy termination in the second trimester and desired fertility preservation. Multiple adjuvant treatment modalities, either alone or in combination, may help to promote the passing or absorption of the implanted placenta under close monitoring.

## Background

Placenta accreta spectrum (PAS) disorders are rare but dangerous pathological conditions in which the placenta abnormally invades the myometrium [1, 2], other adjacent organs (such as the bladder and rectum) and rarely, the broad ligament [3]. The incidence of PAS disorders has been increasing and seems to parallel the increasing cesarean delivery rate [4]. In clinical practice, the number of pregnancy terminations in the second trimester with concurrent PAS disorders is also increasing. Rashbaum et al. [5] reported that the incidence of placenta accreta encountered during dilation and evacuation (D&E) in the second trimester was 0.04% in 1995, similar to that in the third trimester. The frequency was much higher (2.3%) in the study reported by Morotti and his colleagues in 2012 [6]. According to several case reports, massive hemorrhaging [7], spontaneous uterine rupture [8] or subsequent hysterectomy [9] may occur during second trimester pregnancy termination with PAS disorders, similar to term delivery with PAS disorders. Nevertheless, many patients with PAS disorders who underwent pregnancy termination in the second trimester have a strong desire for future pregnancy. Hysterectomy is not easily accepted in this patient group, and damage to the endometrium should thus be avoided. These unique clinical features of pregnancy termination in the second trimester with concurrent PAS disorders place obstetricians in a complex and delicate situation.

However, there are limited data on pregnancy termination in the second trimester with concurrent PAS disorders in the literature. Specific management strategies for this patient group have seldom been reported, and their efficacy remains unevaluated. Therefore, the present study analyzed 29 patients with PAS disorders who underwent pregnancy termination in the second trimester. With the aim of investigating the optimal management strategies for this condition, our experience and a review of the related literature are discussed.

## Methods

The medical records of all patients with PAS disorders who underwent pregnancy termination in the second trimester (from 13 to 27^+ 6^ weeks of gestation) and received care at Beijing Chao-Yang Hospital between December 2005 and December 2015 were collected and analyzed. This study received ethics approval (ethics approval number: 2016-K-82) from the Medical Ethics Committee of Beijing Chao-Yang Hospital, and written informed consent was provided by the participants. The following information was recorded: clinical and obstetric characteristics of the patients, treatment modality, maternal morbidity, clinical outcomes, subsequent fertility, and other information.

### Diagnosis

The pathological “gold standard” diagnosis of PAS disorders, such as when a biopsy is obtained from an area without invasion or with minor penetration, is not always possible [10]. We preferred to make the final diagnosis according to the patients’ clinical, surgical, and pathological characteristics. Prenatal suspicion of a diagnosis of PAS disorders was based on the presence of high-risk factors related to this disease and imaging evidence (ultrasound and/or magnetic resonance imaging [MRI] findings), and was confirmed by postpartum clinical manifestations and/or pathological examination. Ultrasound features included the retained placenta with myometrial thinning, lacunar spaces, loss of the retroplacental clear space and/or increased vascularity. MRI features included the retained placenta with dark intraplacental bands, placental heterogeneity, placental or uterine bulging, focal interruption of the myometrium and/or periuterine extension of the placenta [11]. Postnatal diagnosis was based on clinical manifestations, imaging evidence, and pathological diagnosis. High-risk factors mainly included placenta previa, previous cesarean delivery, other uterine surgery resulting in myometrial tissue damage, and uterine anomalies [12, 13]. Direct chorionic villi contact with the myometrium and an absence of decidua were the criteria for a histological diagnosis of PAS disorders [14]. The clinical manifestations [15, 16] included the following: [1] difficult manual or piecemeal removal of placental tissue after fetal delivery with or without massive hemorrhaging; and [2] visualization of the placenta outside the uterus, inability to remove the placenta from the uterus, or heavy bleeding from the implantation site after placenta removal during hysterotomy.

### Management strategies

The primary outcome was uterus preservation, and the secondary outcome was complications associated with conservative management. The optimal management strategy was defined as preservation of the uterus without severe complications associated with conservative management. For patients with prenatal suspicion of a diagnosis of PAS disorders, prophylactic uterine artery embolization (UAE) was completed prior to pregnancy termination when there was a risk of severe hemorrhaging and the fetus had a low chance of survival. The method of pregnancy termination in the second trimester included hysterotomy and medical methods. The former was undertaken in patients with PAS disorders and total placenta previa with a previous cesarean delivery or life-threatening heavy hemorrhaging during the induction of labor. Rivanol and mifepristone combined with misoprostol were used for medical pregnancy termination. Uterine artery ligation and intrauterine balloon tamponade were used during hysterotomy to decrease blood loss. If heavy hemorrhaging, uterine rupture, or other life-threatening situations occurred, a hysterectomy was performed to save the patient’s life. For patients with a postnatal diagnosis, when difficult manual or piecemeal removal of placental tissue occurred, forced removal was avoided because of the possibility of serious hemorrhaging.

Regardless of prenatal or postnatal diagnosis and vaginal delivery or delivery by hysterotomy, the implanted placenta was partly or completely retained in situ instead of being forcibly removed. This procedure was defined as conservative management [16], which was applied with informed consent from the patient and under close monitoring. To promote the passing of the implanted placenta and reduce the risk of heavy hemorrhaging and infection, we prescribed certain adjuvant treatments, including UAE, methotrexate (MTX) chemotherapy, traditional Chinese medicine and mifepristone, followed by curettage under ultrasound guidance. UAE was used to prevent massive hemorrhaging, thus avoiding a hysterectomy. MTX chemotherapy (1 mg/kg) was administered intramuscularly on days 1, 3, and 5 every 2 weeks. Folinic acid was given on days 2, 4, and 6 at 10 to 20% of the MTX dose to reduce toxicity. If a patient’s response to MTX was insufficient (i.e., beta-human chorionic gonadotropin [β-HCG] values decreased slowly, and/or blood flow was abundant between the implanted placenta and the uterine wall), another cycle of MTX treatment was recommended with a 2-week interval. In addition, a single dose (50 mg/m^2^) of MTX could be administered via transcatheter infusion into the bilateral uterine arteries before UAE with sponge particles. Mifepristone (50 mg twice daily for 2 to 3 days) and traditional Chinese medicine were also administered as adjuvant treatments to accelerate the expulsion of the residual placenta. The traditional Chinese medicine used in our series is a classical herbal formula named Sheng Hua Tang, comprising Radix Angelica sinensis, Ligustici rhizoma, Semen Persicae, Zingiberis rhizoma, and Radix glycyrrhizae. In China, Sheng Hua Tang is frequently used to treat subinvolution of the uterus and placenta remnants, with the potential mechanism of activating blood flow and removing blood stasis [17–19].

Patients were closely monitored during the adjuvant treatment period. Prophylactic intravenous or oral antibiotics were administered to prevent infection-related complications. Serum β-HCG levels, routine blood measurements, blood coagulation tests, biochemical tests, infection markers (e.g., C-reactive protein (CRP), procalcitonin (PCT), blood culture and vaginal secretion culture) and other tests were assessed once per week. Serial sonographic examinations were performed every 2 weeks to evaluate blood flow between the implanted placenta and myometrium as well as the volume of the residual placenta. If the serum β-HCG values decreased to normal or near normal (less than 5 mIU/ml) and a significant reduction in placenta volume was not observed, curettage under ultrasound guidance was planned in advance to shorten the treatment period, stop irregular vaginal bleeding, promote menstruation recovery, and relieve the psychological burden in patients.

### Statistical analyses

Data were entered and analyzed in SPSS (IBM Corp, Armonk, NY, USA) for Windows 22.0. The results are presented as the mean and standard deviation for quantitative variables and number (percentages) for qualitative variables.

## Results

### Patient characteristics

**Fig. 1:**
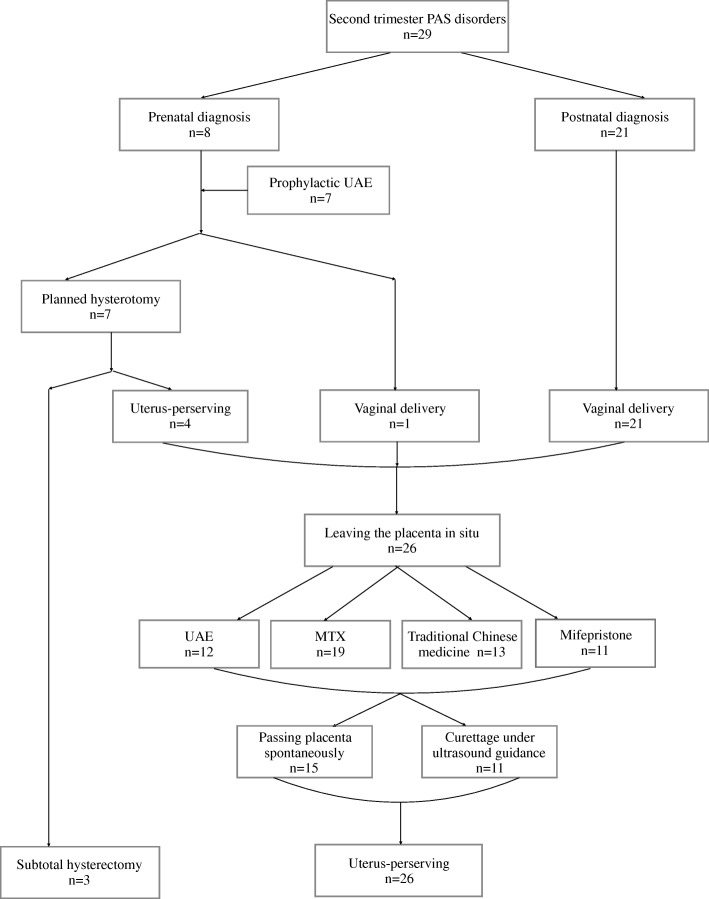
Management strategies and clinical outcomes of patients with placenta accreta spectrum disorders who underwent pregnancy termination in the second trimester

During the study period, 97 consecutive patients with PAS disorders were treated at our hospital. Twenty-nine of these patients (29.9%) underwent pregnancy termination or spontaneous labor in the second trimester. Four (13.8%) patients spontaneously delivered with retained placenta. The compelling reasons for pregnancy termination in the remaining 25 patients included the following: missed abortion or intrauterine fetal death (6 patients, 20.7%), voluntary pregnancy termination in agreement with our national law (8 patients, 27.6%), and fetal malformation (11 patients, 37.9%). A prenatal diagnosis was suspected in 8 patients (27.6%), all of whom had placenta previa (8 patients) and/or a previous cesarean delivery (7 patients). The remaining 21 patients (72.4%) with PAS disorders were diagnosed after delivery of the fetus. All diagnoses were confirmed by postpartum clinical manifestations or pathological examination. The median duration of follow-up was 47 (range: 6 to 126) months. The clinical and obstetric characteristics of the 29 patients are listed in Table 1. The mean patient age was 29.89 (range: 20 to 40) years. The average gestational week at pregnancy termination was 19.36 (range: 13 to 27^+ 4^) weeks. The median gravidity was 2, including 3 nulliparous patients (10.3%). The most common risk factor was previous curettage operation (75.9%), followed by previous cesarean delivery (69.0%) and placenta previa (41.4%). The management modalities and clinical outcomes of the target population are shown in Fig. 1.

**Table 1:** Characteristics of the 29 patients complicated with morbidly adherent placenta who suffered from second trimester abortion

	Mean/Median or Number	Range or Percentage
Age (years) (mean;range)	29.89 ± 5.49	20–40
Gestation weeks at diagnosis (weeks) (mean/range)	19.36 ± 4.56	13–27^+ 4^
Gravidity (median/range)	2	0–4
Parity (median/range)	1	0–2
Prenatal diagnosis (n) (percent)	8	27.6%
Postnatal diagnosis (n) (percent)	21	72.4%
Risk factors (n) (percent)		
Older maternal age (≥ 35 years old)	8	27.6%
Previous curettage	22	75.9%
Previous caesarean delivery	20	69.0%
Once	14	48.3%
Twice	6	20.7%
Placenta previa	12	41.4%
Low-lying placenta	4	13.8%
Marginal placenta previa	4	13.8%
Total placenta previa	7	24.1%
Myomectomy	1	3.4%
Uterine malformation	2	6.9%
Uterus bicornis	1	3.4%
Uterus didelphys	1	3.4%
Twin pregnancy	1	3.4%

In the subgroup with prenatal diagnosis, one patient (1/8, 12.5%) underwent medical termination after preoperative UAE. The fetus was delivered smoothly, and the implanted placenta remained partly in situ with 100 ml of blood loss after partial placenta removal was undertaken. A planned hysterotomy was performed in the remaining 7 patients (7/8, 87.5%) who had total placenta previa and previous cesarean delivery, and preoperative UAE was used for 6 of patients. After the fetuses were delivered and partial placental abruption was resected with suture and ligation, the implanted placenta remained partly or completely in situ because it was difficult to remove from the uterine wall. During hysterotomy, intraoperative bilateral uterine arteries ligation (7 cases) and intrauterine balloon tamponade (4 cases) were performed to attenuate severe hemorrhaging. Massive hemorrhage occurred in all 7 patients, and 3 of the patients developed disseminated intravascular coagulation (DIC). The average estimated blood loss was 2474.2 (range: 800 to 6630) ml. Six patients required a blood transfusion. Unfortunately, subtotal hysterectomy was performed in 3 (3/29, 10.3%) patients with life-threatening bleeding and a poor response to multiple hemostatic measures. Moreover, one of these patients underwent simultaneous partial bladder resection and repair surgery because of bladder invasion. The uterus was preserved with an in situ placenta in the 4 remaining patients. In the subgroup with a postnatal diagnosis, the placenta could not be spontaneously passed after the fetus was delivered in all 21 patients. After manual removal of the placenta failed, the implanted placenta remained partly or completely in situ in all 21 patients under informed consent. The average estimated blood loss was 616.1 (range: 85 to 2200) ml, and the number of patients with more than 500 ml of blood loss was 8. Seven patients required a blood transfusion.

Thus, a total of 26 (26/29, 89.7%) patients partially or completely retained the implanted placenta in situ in the whole series. The implanted placenta was present in a uterine scar in 4 (15.4%) of these patients. Adjuvant treatments, including UAE and medication, as well as curettage were administered alone or in combination to the 26 patients with an in situ placenta. Adjuvant treatment details are listed in Table 2. UAE was performed in 12 (46.2%) patients after termination. MTX chemotherapy was administered to 19 (73.1%) patients, including 6 (23.1%) who received a transcatheter infusion of MTX into the bilateral uterine arteries before embolization with sponge particles. Nine (34.6%) patients received repeated cycles of MTX chemotherapy; the maximum number of cycles was 3 (1 patient, 3.8%). Traditional Chinese medicine and mifepristone were administered to 13 (50%) and 11 (42.3%) patients, respectively. The residual placenta was spontaneously passed through the vagina in 15 (57.7%) patients. For the remaining 11 (42.3%) patients, curettage under ultrasound guidance was performed to remove the residual placenta. In 1 (3.8%) patient, laparoscopy was used in combination with ultrasound as another monitoring modality. Four (15.4%) patients underwent UAE prior to curettage to prevent massive hemorrhaging during the operation. The range of serum levels of β-HCG was 0 to 65.83 mIU/ml when curettage was performed. The mean curettage operation duration was 32 (range: 20 to 70) minutes, and the average blood loss was 113.0 (range: 10 to 400) ml. The mean decrease in hemoglobin was 0.48 (range: 0 to 1.30) g/dl.

**Table 2:** Adjuvant treatment details of the 26 patients who retained the implanted placenta in situ

	Number	Percentage %
UAE^a^	12	46.2
MTX^b^ chemotherapy	19	73.1
Traditional Chinese medicine	13	50.0
Mifepristone	11	42.3
Curettage^c^	11	42.3

^a^UAE, uterine artery embolization; ^b^MTX, methotrexate; ^c^Curettage, followed by medication treatment under ultrasound guidance

Among the 26 patients who underwent conservative management, 2 (7.7%) experienced delayed postpartum hemorrhage requiring transfusion, with blood loss ranging from 600 to 1300 ml. Other complications associated with conservative management included fever (6 cases, 23.1%), G1 transaminase disorder (4 cases, 15.4%), and G2 myelosuppression (1 case, 3.8%) according to the Common Terminology Criteria for Adverse Events version 4.0. The average interval between the date of fetal delivery and complete passing of the placenta as determined by ultrasound examination was 76.6 (range: 19 to 192) days. The time required for serum β-HCG values to decrease to a normal level was 52.6 (range: 15 to 109) days. Regular menstruation resumed 2.5 (range: 1 to 5) months after fetal delivery. Seven (26.9%) women had a spontaneous pregnancy after conservative management, and 6 women had full-term vaginal delivery (2 patients) or cesarean delivery (4 patients). No patient experienced recurrent PAS disorders.

## Discussion

It is very important yet difficult to treat second trimester PAS disorders because of the difficulties of prenatal diagnosis and the patient’s strong desire for future fertility, which creates a dilemma for obstetricians. Prenatal diagnosis of PAS disorders is crucial; based on prenatal diagnosis, appropriate multidisciplinary clinical decisions can minimize potential maternal morbidity and mortality [20]. In the present study, 27.6% of the patients with PAS disorders received a suspected diagnosis before pregnancy termination in the second trimester through initial ultrasound and MRI screening, and all of these patients had placenta previa and/or previous cesarean delivery. For high-risk patients, MRI was often performed when PAS disorders were suspected based on the initial ultrasound examination [21]. MRI might be more accurate than ultrasound if PAS disorders are suspected in a posterior placenta or in patients with obesity [22]. MRI has high predictive accuracy in assessing the depth and topography of placental invasion [23], which can help doctors determine management strategies. At our hospital, among patients with a suspected prenatal diagnosis of PAS disorders, vaginal delivery after medically induced pregnancy termination was the preferred methods of management considering the low probability of fetal survival in the second trimester for those women desiring future fertility. Hysterotomy was performed only in patients with PAS disorders with total placenta previa and previous cesarean delivery or in those who experienced life-threatening heavy hemorrhaging during the induction of labor.

If the implanted placenta cannot be separated from the uterine wall, leaving the placenta in situ may be a wise decision in patients with stable hemodynamics and no life-threatening bleeding [24]; this management strategy was supported by our series. However, leaving the placenta in situ may lead to infection, delayed hemorrhaging, secondary hysterectomy, and potential complications from adjuvant treatments. Therefore, close surveillance, including various tests and imaging examinations, is necessary to detect complications in a timely manner. In our series, the placenta remained in situ in 26 patients, and the uterus was successfully preserved in all patients without severe complications or maternal death. Seven spontaneous pregnancies occurred after PAS disorders; however, the impact of conservative management on subsequent pregnancy needs to be evaluated by long-term follow-up to determine the safety and efficacy of this management strategy.

In our series, UAE, MTX chemotherapy, traditional Chinese medicine, and/or mifepristone followed by curettage under ultrasound guidance were viable adjuvant treatments in the fertility-preserving approach. A systematic review of uterine-preserving approaches suggested that UAE was a safe and effective treatment for postpartum hemorrhaging caused by PAS disorders [25]. Yu et al. [26] reported that prophylactic intraoperative UAE before placenta involution appeared to prevent postpartum hemorrhage during late gestation. In our series, prophylactic UAE was performed in 7 patients, and the uterus was preserved in 5 patients without embolization complications. Based on our experience, prophylactic UAE before termination is a necessary and effective procedure, especially for patients with PAS disorders with total placenta previa and previous cesarean delivery. In our series, MTX therapy, traditional Chinese medicine, and/or mifepristone were used to promote the passing of the implanted placenta. However, the administration of these medicines in the treatment of PAS disorders remains unclear and controversial. Morgan et al. [27] reported that mifepristone and misoprostol were used successfully in patients with PAS disorders at term. Some experts have used MTX therapy for PAS disorders and suggested that MTX therapy was effective against trophoblastic proliferation [28, 29]. However, there is no standard recommended dosage of MTX for PAS disorders, and the mode of administration, including in situ, intramuscular, intraumbilical and uterine artery infusion, varied widely according to the authors [30]. Meanwhile, some studies and systematic reviews did not recommend the use of MTX therapy for conservative management [31–33]. The role of MTX in treating PAS disorders remains controversial because of its uncertain function and possible side effects, and additional evidence of its efficacy and safety is required [16]. In addition, immunosuppression, gastrointestinal complications, pancytopenia, hepatotoxicity, and nephrotoxicity may be observed with adjunctive medication treatments [19]. In the present study, transaminase disorder and myelosuppression were observed, but all patients completely recovered with symptomatic treatment. We could not draw any definitive conclusions due to the small sample size, and the risks and benefits of the adjunctive medication treatments must be tested and verified in additional studies. Serum β-HCG values returning to normal despite a large residual placenta remaining in the uterine cavity after medication, implies that the placenta is no longer functioning [34]; in that case, curettage under ultrasound guidance is usually recommended. In the present study, curettage was performed in 42.3% of patients, and all operations went smoothly with little bleeding. According to our experiences and the data of the present study, curettage under ultrasound guidance could be planned in advance if the serum β-HCG values decreased to normal or near normal levels. However, because of the small sample size and lack of control groups and statistical analysis, we cannot specify a definite threshold of the serum levels of β-HCG. The appropriate timing of curettage can be determined from blood flow between the implanted placenta and uterine wall under ultrasound scanning and serum levels of β-HCG [19]. In addition, UAE and/or laparoscopy can be used in combination with curettage. Consequently, the management strategies for patients with PAS disorders who undergo pregnancy termination in the second trimester should be comprehensive and individualized. Patients should be informed of the risks, benefits and alternative treatment options. Blood should be available for possible transfusion and the cooperation of multiple services that may be necessary should be assured.

As the present study was retrospective and included a small sample size, the results and conclusions should be interpreted with caution. In particularly, the lack of comparison of different adjuvant treatment groups and statistical analysis may have interfered with the results. A randomized trial will better compare the advantages and disadvantages of the different management strategies; however, it is difficult to perform such trials on this life-threatening condition. The present study included 29 cases of second trimester PAS disorders and is one of the largest published series. In addition, this analysis spanned the past 10 years, reflecting the latest treatment outcomes of this disease.

## Conclusions

Our preliminary results suggest that leaving the implanted placenta in situ appears to be the preferred choice for patients with PAS disorders who underwent pregnancy termination in the second trimester and desire fertility preservation. Multiple adjuvant treatment modalities, either alone or in combination, may help to promote the passing or absorption of the implanted placenta under close monitoring.

## Abbreviations

CRP: C-reactive protein
D&E: Dilation and evacuation
DIC: Disseminated intravascular coagulation
MRI: Magnetic resonance imaging
PAS: Placenta accreta spectrum
PCT: Procalcitonin
UAE: Uterine artery embolization
β-HCG: Beta-human chorionic gonadotropin
